# Avant-propos de l’Association Internationale de la Mutualité (AIM)

**DOI:** 10.48327/mtsimagazine.n1.2021.77

**Published:** 2021-04-02

**Authors:** S. Reichert, T. Kanga-Tona

**Affiliations:** Association Internationale de la Mutualité - Rue d’Arlon 50 B-1000, Bruxelles, Belgique

C’est avec un grand honneur et un grand plaisir que le mouvement mutualiste se voit être mis en valeur dans le *focus* de ce numéro de l’année 2021 de la Revue de la Société Francophone de Médecine Tropicale et Santé Internationale (SFMTSI). Les mutuelles de santé sont des associations à but non lucratif qui se donnent pour mission de protéger leurs membres contre les dépenses liées à l’accès aux soins de santé. Les mutuelles remplissent également d’autres missions telles que la prévention et la promotion de la santé, ou l’offre de services qui agissent sur les déterminants sociaux de la santé. Organisées au niveau professionnel (mutuelles des agents des différentes fonctions publiques par exemple), elles sont également présentes au niveau communautaire en zone rurale.

Les mutuelles facilitent pour leurs membres l’accès aux services de santé à travers les mécanismes de solidarité. Elles fonctionnent sur base d’un mécanisme de partage des risques et de mise en commun des ressources. Les décisions concernant l’organisation des prestations des services sont prises par les organes de gouvernance des mutuelles qui réunissent les membres. Les mutuelles sont des véhicules du progrès social et s’engagent notamment pour l’égalité hommes-femmes au sein des structures militantes, auprès des adhérents mutualistes et dans la vie des instances. Elles exercent donc leur activité au plus près des besoins de leurs membres suivant une logique participative et démocratique. Contrairement aux assurances privées, les mutuelles de santé n’opèrent aucune sélection des membres liée au risque individuel.

L’action et les caractéristiques des mutuelles leur permettent d’agir sur les trois objectifs de la couverture de santé universelle :

- L’accès équitable aux services de santé - tous ceux qui ont besoin des services de santé, quels que soient leurs moyens financiers, doivent pouvoir y accéder;
- La qualité - les services de santé doivent être d’une qualité suffisante pour améliorer la santé de ceux qui en bénéficient;
- La protection financière - le coût des soins ne doit pas exposer les usagers à des difficultés financières.

Les mutuelles aident également à atteindre un certain nombre d’Objectifs de Développement Durable (ODD) de réduction de la pauvreté, d’égalité des sexes, de croissance soutenable, ainsi que de travail décent et de réduction des inégalités entre les pays.

Au cours des vingt dernières années, le mouvement mutualiste a connu une dynamique de structuration régionale et nationale en Afrique. Cette vague, qui s’est concrétisée par la création de plateformes mutualistes représentatives au sein de pays, s’est appuyée sur une augmentation de leurs compétences en termes de gestion opérationnelle de la couverture maladie mais aussi sur la formation, l’adoption et/ou la mise en œuvre de textes réglementaires, la recherche scientifique, l’organisation d’évènements. Ces développements font des mutuelles de santé des partenaires essentiels pour le développement des couvertures de santé universelle en Afrique.

Les mutuelles sont réunies au niveau international au sein de l’Association Internationale de la Mutualité (AIM). L’AIM est en charge d’animer le mouvement mutualiste ainsi que d’en assurer la visibilité auprès des institutions internationales et européennes. L’AIM propose ainsi dans l’ensemble des articles qui seront publiés en ligne dans la partie magazine de la revue Médecine Tropicale et Santé Internationale: un plaidoyer pour la promotion des mutuelles en Afrique; un zoom sur les actions du programme Mon Assurance Santé Mutualiste (MASMUT) et du Programme d’Appui aux Stratégies Sociales (PASS). Nous présenterons ensuite une avancée mutualiste spécifique à l’Afrique de l’Ouest, qui est le code de la Mutualité, adopté par l’Union Economique et Monétaire Ouest-Africaine (UEMOA). L’UEMOA propose de réglementer régionalement l’activité des mutuelles de santé. Enfin, nous offrirons un aperçu du rôle des mutuelles dans la lutte contre la COVID-19 dans de nombreux pays d’Afrique. Nous avons synthétisé ce rôle et nos demandes durant cette période au sein d’une déclaration officiellement adoptée par le mouvement mutualiste en novembre 2020.

